# Supplementary material for: PASCAR: a multiscale framework to explore the design space of constitutive and inducible CAR T cells
Source: Life Sci Alliance. 2023 Jul 28;6(10):e202302171. doi: 10.26508/lsa.202302171 (PMC10387492; doi:10.26508/lsa.202302171)
Supplement: Supplementary file 1 [file LSA-2023-02171_Supplemental_Data_1.pdf]

Derivation of Equations (1) and (2)

The ODEs describing the mass action kinetics for species abundances C_0, C_1, …., C_N for the reaction scheme shown in Fig. 1C are given by,

$$\frac{dC_0}{dt} = k_{on}[R][H] - (k_{off}+k_p)\, C_0$$
$$\frac{dC_1}{dt} = k_p C_0 - (k_{off}+k_p)\, C_1$$

.

$$\frac{dC_{N-1}}{dt} = k_p C_{N-2} - (k_{off}+k_p)\, C_{N-1}$$
$$\frac{dC_N}{dt} = k_p C_{N-1} - k_{off}\, C_N$$

The total numbers of R and H are fixed and are given by R_0 and H_0, respectively. The conservation of R_0 and H_0 relates the number of free R and H, denoted by [R] and [H], respectively, to C_0, C_1, …., C_N as,

$$[R] = R_0 - (C_0 + C_1 + \cdots + C_N) \qquad \text{(i)}$$

$$[H] = H_0 - (C_0 + C_1 + \cdots + C_N) \qquad \text{(ii)}$$

At steady state, $\frac{dC_0}{dt} = \frac{dC_1}{dt} =..= \frac{dC_N}{dt} = 0$, and the abundances of the species C_1 to C_N at the steady state are given by,

$$C_1 = \frac{k_p}{k_p + k_{off}} C_0 = \alpha C_0$$

where, $\alpha = \frac{k_p}{k_p+k_{off}}$.

$$C_2 = \alpha C_1 = \alpha^2 C_0$$

….

$$C_{N-1} = \alpha^{N-1} C_0$$
$$C_N = (\frac{k_p}{k_{off}})\alpha^{N-1} C_0$$

The total number of the complexes $C_{total} = C_0 + C_1 + \cdots + C_N$ is given by,

$$C_{total} = C_0 \left(1 + \alpha + \alpha^2 \ldots + \alpha^{N-1} + \left(\frac{k_p}{k_{off}}\right)\alpha^{N-1}\right)$$
$$= C_0\left(\frac{1-\alpha^N}{1-\alpha} + \left(\frac{k_p}{k_{off}}\right)\alpha^{N-1}\right) = C_0\left(\frac{1-\alpha^N}{1-\alpha} + \frac{\alpha}{1-\alpha}\alpha^{N-1}\right) = \frac{1}{1-\alpha} C_0$$

We further simplify the expressions by introducing a parameter β, where,

$$\beta = \frac{1}{1-\alpha} = \frac{k_p + k_{off}}{k_{off}} = 1 + k_p/k_{off}$$

The expression for C_N, can be simplified further by writing it in terms of C_{total},

$$C_N = \left(\frac{k_p}{k_{off}}\right)\alpha^{N-1}C_0 = \left(\frac{\alpha}{1-\alpha}\right)\alpha^{N-1}C_0 = \alpha^N C_{total}$$

Eqs.(i)-(ii) can be further simplified to,

$$[R] = R_0 - \beta C_0$$
$$[H] = H_0 - \beta C_0$$

The steady state condition for $\frac{dC_0}{dt} = 0$ yields,

$$k_{on}(R_0 - \beta C_0)(H_0 - \beta C_0) - (k_{off} + k_p)\, C_0 = 0$$

$$\Rightarrow\ \beta^2 C_0^2 - \left(\beta R_0 + \beta H_0 + K_D + \left(\frac{k_p}{k_{on}}\right)\right) C_0 - R_0 H_0 = 0$$

$$\Rightarrow\ {C_0}^2 - \left(\frac{R_0}{\beta} + H_0/\beta + (K_D + \left(\frac{k_p}{k_{on}}\right))/\beta^2\right) C_0 - R_0 H_0/\beta^2 = 0$$

Define, $R_0' = \frac{R_0}{\beta}, H_0' = \frac{H_0}{\beta}, K_D' = \frac{K_D + \left(\frac{k_p}{k_{on}}\right)}{\beta^2}$. The above equation in terms of the new variables is then,

$${C_0}^2 - (R_0' + H_0' + K_D')C_0 - R_0' H_0' = 0$$

The solution is given by,

$$C_0 = \frac{1}{2}(R_0' + H_0' + K'_D)\left(1 - \sqrt{1 - \frac{4R_0' H_0'}{\left(R_0' + H_0' + K_D'\right)^2}}\right)$$

We can write the above equation as,

$$C_0 = \frac{1}{2\beta}(R_0 + H_0 + \beta K'_D)\left(1 - \sqrt{1 - \frac{4R_0 H_0}{\left(R_0 + H_0 + \beta K_D'\right)^2}}\right)$$

or

$$C_0 = \frac{1}{2\beta}\left(R + H + \widetilde{K}_D\right)\left(1 - \sqrt{1 - \frac{4RH}{\left(R + H + \widetilde{K}_D\right)^2}}\right) \qquad \text{(iii)}$$

where, $\widetilde{K}_D = \beta K'_D = (K_D + k_p/k_{on})/\beta$

Substituting $\beta = \frac{k_p + k_{off}}{k_{off}}$ in the expression $\widetilde{K}_D$ of yields,

$$\widetilde{K}_D = \beta K'_D = \frac{K_D + \left(\frac{k_p}{k_{on}}\right)}{\beta} = \frac{k_{off}\, K_D + \frac{k_{off} k_p}{k_{on}}}{k_p + k_{off}} = \frac{k_{off}\, K_D + k_p K_D}{k_p + k_{off}} == \left(\frac{k_{off} + k_p}{k_p + k_{off}}\right) K_D = K_D$$

Therefore, we can write Eq. (iii) as,

$$C_0 = \frac{1}{2\beta}(R_0 + H_0 + K_D)\left(1 - \sqrt{1 - \frac{4R_0 H_0}{(R_0 + H_0 + K_D)^2}}\right)$$

Now, we will use the relation below for C_N

$C_N = \left(\frac{1}{1-\alpha}\right) \alpha^N C_0 = \beta \alpha^N C_0.$
