# Supplementary material for: PASCAR: a multiscale framework to explore the design space of constitutive and inducible CAR T cells
Source: Life Sci Alliance. 2023 Jul 28;6(10):e202302171. doi: 10.26508/lsa.202302171 (PMC10387492; doi:10.26508/lsa.202302171)
Supplement: Supplementary file 2 [file LSA-2023-02171_Supplemental_Data_2.pdf]

Effect of diffusion on the receptor-ligands binding/unbinding rates

Consider binding and unbinding of the receptors R with ligands H. R and H diffuse on the plasma membranes of T cell and target cells with diffusion constants D(H) and D(R), respectively. Following Eigen(Eigen, 1974), Bell(Bell, 1978), Keizer(Keizer, 1987), and others, we consider R and H molecules approach each other by diffusion and interact when they are within small distance *d* (= 2 nm). In this method, binding unbinding reactions (H+R ↔ HR) given by binding rate k_{on} and unbinding rate k_{off} are described in two steps where H and R approach each other within *d* to form an encounter complex H:R and then react to form the complex HR. The rates for these reactions are described below:

$$H + R \underset{d_-}{\overset{d_+}{\rightleftarrows}} H{:}R \underset{r_-}{\overset{r_+}{\rightleftarrows}} HR$$

The mass action kinetics for the above reactions are given by,

$$\frac{d[H]}{dt} = d_+[H][R] - d_-[H{:}R]$$
$$\frac{d[R]}{dt} = d_+[H][R] - d_-[H{:}R]$$
$$\frac{d[H{:}R]}{dt} = d_+[H][R] - (d_- + r_+)[H{:}R] + r_-[HR]$$
$$\frac{d[HR]}{dt} = r_+[H{:}R] - r_-[HR]$$

The rates d_+ and d_- are given by in three dimensions as,

$d_+ = 4\pi(D(H) + D(R))d$ (ia)

$d_- = 3(D(H) + D(R))/d^{-2}$ (ib)

and in two dimensions as,

$d_+ = 2\pi(D(H) + D(R))$ (iia)

$d_- = 2(D(H) + D(R))/d^{-2}$. (iib)

Assuming a steady state for [H:R], i.e., d[H:R]/dt=0, which is a reasonable approximation in this case, the above equations are reduced to

$$\frac{d[H]}{dt} = k_{on}[H][R] - k_{off}[H{:}R]$$
$$\frac{d[R]}{dt} = k_{on}[H][R] - k_{off}[H{:}R]$$

$$\frac{d[HR]}{dt} = k_{on}[H][R] - k_{off}[HR]$$

where,

$$k_{on} = \frac{r_+ d_+}{d_- + r_+} \quad \text{(iii)}$$

$$k_{off} = \frac{r_- d_-}{d_- + r_+} \quad \text{(iv)}$$

The binding (k_{on}) and unbinding (k_{off}) rates are usually measured in surface plasmon resonance (SPR) experiments, where the receptor molecules are plate bound and the ligands molecules diffuse in three dimensions in the solution.

High Affinity binding:

The values of k_{on} and k_{off} values for WT sequences were measured by biolayer interferometry sensograms by Hernandez-Lopez et al. (Fig. S2B in their manuscript). The reported affinities for the high affinity CAR are given by,

$k_{on} = 5.1 \times 10^3\ M^{-1}s^{-1}$
$k_{off} = 9.0 \times 10^{-5}\ s^{-1}$
$K_D = 17.6$ nM

We use the above values, the diffusion constants, and Eqs. (i)-(iv) to calculate r_+ and r_- values for the reactions.

We assume D(H) = 10 μm^2/s, D(R) = 0 (immobilized on the surface), and d=2nm. Thus, d_+ and d_- are calculated using Eqs (i)(a-b), as

$d_+ = 4\pi D(H) d \approx 12\times 10\ \mu m^2 s^{-1} \times 2nm = 240 \times 10^{-3}\ \mu m^3/s$
and
$d_- = 3D(H)/d^2 = 3\times 10\ \mu m^2 s^{-1} / 4nm^2 = 7.5 \times 10^6$ /s .

The above d_+ and d_- values are used to calculate r_+ and r_- following,

$$r_+ = \frac{k_{on} d_-}{d_+ - k_{on}} \approx 5.1 \times 7.5 \times 10^9/(7.5 \times 10^6)\ s^{-1} = 2.5 \times 10^2\ s^{-1} \quad \text{(v)}$$

$$r_- = \frac{k_{off}\,(d_- + r_+)}{d_-} = \frac{9.0\times10^{-5}(7.5\times10^6+250)}{7.5\times10^6} s^{-1} \approx 9.0 \times 10^{-5} s^{-1} \quad \text{(vi)}$$

In our model, R and H molecules are membrane bound and diffuse at a smaller rate than in the solution. We assume D(R)=D(H)=0.01 μm^2/s in the membrane, and then use Eqs. (iii)-(iv) and the r_+ and r_- values in Eqs. (v) and (vi) to calculate the effective k_{on} and k_{off} rates for this system. Note, the reaction rate r_+ in Eq. (v) is about 16 times smaller than of the two dimensional diffusion rate,

$$d_- = \frac{2(D(H)+D(R))}{d^2} = \frac{2 \times 0.02\ \mu m^2 s^{-1}}{(4\ nm)^2} = 10^4 s^{-1},$$

therefore, the k_{on} and k_{off} values are going to be changed by a small amount here.

The values of k_{on} and k_{off} obtained from Eqs. (iii) and (iv) are given by,

$$k_{on} = \frac{(0.12 \times 250)\mu m^2 s^{-2}}{(10^4+250)s^{-1}} = 5.1\times10^{-3}\ \mu m^2/\text{molecules } s^{-1} \quad \text{(vi)}$$

and,

$$k_{off} = \frac{10^4 \times 9.0 \times 10^{-5}}{10^4 + 250} s^{-1} \approx 9.0 \times 10^{-5} s^{-1}$$

Our calculation where we converted k_{on} values measured in three dimensions to two dimensions as $(k_{on})_{2D}=(k_{on})_{3D}/d$ produces a value of

$k_{on} = 5.1 \times 10^3\ M^{-1}s^{-1}/(2\ nm) = 4.3 \times 10^{-3}\ \mu m^2/$ molecules s^{-1}

which is about a 16% decrease from the value in Eq. (vi).

Low Affinity binding:

The reported affinities for the how affinity CAR in Hernandez-Lopez et al. are given by,

$k_{on} = 3.2 \times 10^4\ M^{-1}s^{-1}$
$k_{off} = 6.8\times 10^{-4}\ s^{-1}$
K_D=210 nM

As described above, we first calculated r_+ and r_- values considering 3D diffusion and then incorporated the effect of 2D diffusion on k_{on} and k_{off} following Eqns. (iii) and (iv). The results are shown below.

$k_{on} = 3.4\times10^{-3}\ \mu m^2/$molecules s^{-1} (vii)
$k_{off} \approx 6.8 \times 10^{-4} s^{-1}$

Our calculation where we converted k_{on} values measured in three dimensions to two dimensions as $(k_{on})_{2D}=(k_{on})_{3D}/d$ produces a value of

$k_{on} = 2.7 \times 10^{-3}\ \mu m^2/$ molecules s^{-1}

which is about a 20% decrease from the value in Eq. (vii).

Bell, G.I. (1978). Models for the specific adhesion of cells to cells: a theoretical framework for adhesion mediated by reversible bonds between cell surface molecules. Science *200*, 618-627.
Eigen, M. (1974). Quantum statistical mechanics in the natural sciences. (Plenum, New York).
Keizer, J. (1987). Diffusion effects on rapid bimolecular chemical reactions. Chemical reviews *87*, 167-180.
