# Supplementary material for: PASCAR: a multiscale framework to explore the design space of constitutive and inducible CAR T cells
Source: Life Sci Alliance. 2023 Jul 28;6(10):e202302171. doi: 10.26508/lsa.202302171 (PMC10387492; doi:10.26508/lsa.202302171)
Supplement: Supplementary file 3 [file LSA-2023-02171_Supplemental_Data_3.pdf]

Estimation of the probability of contact for CAR T cells and target cells in cytotoxic assays in vitro

The co-culture experiments were performed in 96 well tissue culture plates where each well has a volume V (=0.2 mL). For an assay with 10,000 (n_{tcell}) CD8+ T cells and 20,000 (= n_{target}) target cells we estimate the probability of a CD8+ T cell having at least one target cell in contact with it. We divide the volume V in cubic chambers of volume v=(d_{tumor}+ d_{tcell})^3, where d_{tumor} (= 6 µm) and d_{cell} (=17 µm) are the diameters of a K562 cell(Henslee et al., 2011) and a CD8+ T cell, respectively. Thus, the culture well can be divided into V/v (≈ 1.6 × 10^7) small chambers and we assume the target and the CD8+ T cells are distributed uniformly and randomly in the culture well. The probability for choosing a target cell or a CD8+ T cells is $p_{target} = n_{target}/ (V/v) \approx 1.25\times 10^{-3}$, or, $p_{tcell} = n_{tcell}/ (V/v) = 0.625 \times 10^{-3}$. Now we can estimate what is the probability (=$p_{contact}$(n)) that a T cell will share a small chamber with n number of target cells which will be given by,

$$p_{contact}(n) = {}^{n_{target}}C_n(p_{tcell})^n(1 - p_{tcell})^{\,n_{target}-n}$$

Thus, the probability that a CD8+ T cell does not reside in the same chamber with a target cell is $p_{contact}(n = 0) = (1 - p_{tcell})^{n_{target}} \approx 1.9 \times 10^{-3}$. Thus, 99.8% CD8+ T cells are in contact with target cells in the culture well. Variation of $p_{contact}(n)$ with n is shown below.

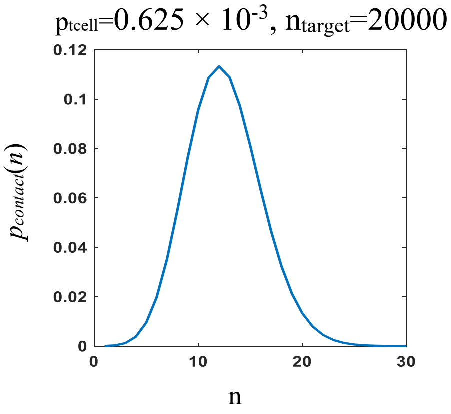

Henslee, B.E., Morss, A., Hu, X., Lafyatis, G.P., and Lee, L.J. (2011). Electroporation dependence on cell size: optical tweezers study. Analytical chemistry *83*, 3998-4003.
